# Dysfunction of Autophagy in Adipose Tissue Macrophages Regulated via FoxO1 in Obesity-Related Severe Acute Pancreatitis

**DOI:** 10.3390/ijms26157206

**Published:** 2025-07-25

**Authors:** Xin Ling, Zewen Zhang, Lihui Lin, Xianwen Guo, Zhen Ding

**Affiliations:** Department of Gastroenterology, The First Affiliated Hospital, Sun Yat-sen University, Guangzhou 510080, China

**Keywords:** obesity-related severe acute pancreatitis, adipose tissue macrophages, autophagic flux, FoxO1, SNARE proteins

## Abstract

Adipose tissue macrophages (ATMs) play important roles in the progression of obesity-related severe acute pancreatitis (SAP). This study aimed to investigate the alterations of autophagic flux within ATMs, as well as the possible regulatory mechanisms. Obese mice were induced via high-fat diets. SAP was triggered using caerulein and lipopolysaccharide. Inflammatory injuries within pancreatic and adipose tissue were assessed. Autophagic flux, along with the expression of autophagosome-located soluble N-ethylmaleimide-sensitive factor attachment protein receptor (SNARE) proteins, were examined in ATMs. RNA-sequencing was performed to identify the possible regulatory factor, which was further validated. The results showed that obesity exacerbated inflammatory injuries. ATMs in obesity-related SAP exhibited impaired autophagic flux characterized by reduced autophagosome–lysosome fusion. Expression of autophagosome-located SNARE proteins decreased in ATMs. RNA-sequencing identified Forkhead box as the differentially expressed transcription factor associated with autophagy. The expression and transcriptional activity of Forkhead box O1 (FoxO1) decreased. The inhibition of FoxO1 exacerbated SNARE proteins’ suppression and autophagic flux impairment, while the activation of FoxO1 showed the opposite effect. In conclusion, obesity-induced impaired autophagic flux and autophagosome–lysosome fusion in ATMs are potentially regulated via autophagosome-located SNARE proteins and the transcription factor FoxO1. The impaired autophagic flux in ATMs aggravated inflammatory injuries of obesity-related SAP.

## 1. Introduction

Severe acute pancreatitis (SAP), characterized by a mortality rate exceeding 20%, is frequently complicated due to systemic inflammatory response syndrome and multiple organ failure [1,2,3]. The global obesity epidemic, driven by contemporary lifestyles, constitutes an established independent risk factor for both the incidence of acute pancreatitis (AP) and disease progression to SAP [4,5]. This clinical correlation implies the existence of a specific obesity-mediated pathophysiological mechanism in SAP development that remains unclear.

Macrophages constitute important regulators in the inflammatory cascade of AP pathogenesis [6,7]. Notably, adipose tissue macrophages (ATMs) exhibit quantitative expansion correlating with obesity progression, positioning them as key contributors to inflammation in obese states [8,9,10]. Our previous research revealed that ATM-derived inflammatory cytokines exacerbate inflammatory injury in obesity-related SAP [11]. However, the precise molecular mechanisms underlying ATM-mediated inflammatory regulation in this disease context require further elucidation.

Impaired autophagy within pancreatic acinar cells has been established as a critical pathophysiological mechanism underlying AP development [12,13], where insufficient autophagic flux contributes to pancreatic damage [14]. Emerging evidence indicates that autophagic dynamics in macrophages exert regulatory control over inflammatory responses, with autophagic deficiency correlating with increased inflammatory cytokine secretion [15]. The last step of autophagy is the fusion of autophagosome with lysosome, which is highly associated with the impaired autophagic flux [16]. This fusion process is mediated via soluble N-ethylmaleimide-sensitive factor attachment protein receptor (SNARE) proteins [17,18]. Among the SNARE proteins, autophagosome-localized Q-SNAREs and lysosome-localized R-SNAREs have been confirmed as essential participants [19]. However, the specific regulation of the autophagosome–lysosome fusion in SAP, particularly within the unique microenvironment of ATMs in obesity context, remains incompletely defined. We hypothesize that obesity-induced autophagic dysfunction in ATMs may critically modulate inflammatory progression.

This study examined the differentially expressed transcription factors in adipose tissue to identify potential regulatory pathways. The autophagic flux dynamics and autophagosome–lysosome fusion in ATMs were focused on. This research aimed to explore the underlying mechanisms linking ATMs to the inflammation of obesity-related SAP.

## 2. Results

### 2.1. ATMs Participated in the Aggravated Inflammation of SAP Caused by Obesity

**Figure 1:**
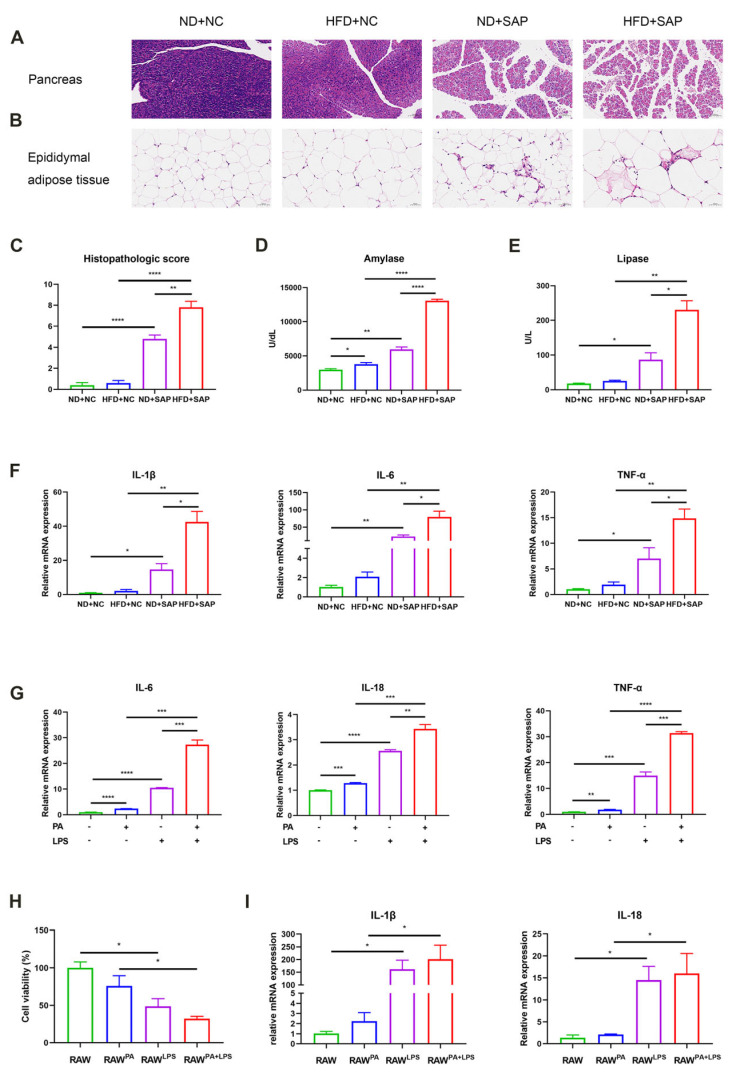
ATMs participated in the aggravated inflammation of SAP caused by obesity. Hematoxylin/eosin (HE) staining of the pancreatic tissue (**A**) (scale: 100 μm) and epididymal adipose tissue (**B**) (scale: 50 μm). (**C**) Histopathologic scores for pancreatic tissue based on edema, acinar necrosis, inflammatory infiltration, hemorrhage, and fat necrosis. Biochemical assays for the levels of serum amylase (**D**) and lipase (**E**). (**F**) The mRNA levels of interleukin (IL)-1β, IL-6, and tumor necrosis factor (TNF)-α in adipose tissue were tested via qPCR. (**G**) The mRNA levels of IL-6, IL-18, and TNF-α in macrophages were examined via qPCR. (**H**) The cell viability of PACs was examined through Cell Counting Kit-8 (CCK-8). RAW^PA^: macrophages pretreated with PA; RAW^LPS^: macrophages pretreated with LPS; RAW^PA+LPS^: macrophages pretreated with PA and LPS. (**I**) The mRNA levels of IL-1β and IL-18 in PACs were examined via qPCR. * *p* < 0.05, ** *p* < 0.01, *** *p* < 0.001, and **** *p* < 0.0001.

A histopathological evaluation of the pancreas and adipose tissue was conducted following SAP induction. Compared with control groups, SAP-induced mice demonstrated significantly aggravated pancreatic inflammatory injury, accompanied by enhanced fat necrosis and inflammatory infiltration in adipose tissue. Notably, the obesity-related SAP mice exhibited more severe inflammatory damage in both the pancreas and adipose tissue than the normal-diet SAP mice (Figure 1A,B). Pancreatic histopathological scoring, based on pancreatic edema, acinar necrosis, inflammatory infiltration, hemorrhage, and fat necrosis, confirmed the tissue damage (Figure 1C). Mice with obesity-related SAP also showed significantly elevated serum amylase and lipase (Figure 1D,E). A quantitative PCR (qPCR) analysis demonstrated upregulated inflammatory cytokines in the adipose tissue of obesity-related SAP mice (Figure 1F). To verify the change in ATMs in vitro, palmitic acid (PA) and lipopolysaccharide (LPS) were applied to stimulate mouse macrophages, which significantly increased inflammatory cytokines (Figure 1G). To further examine the effect of ATMs on the pancreas, the pretreated macrophages were co-cultured with pancreatic acinar cells (PACs). PA/LPS-treated macrophages could inhibit the viability of PACs (Figure 1H) and promote the expression of inflammatory cytokines in PACs (Figure 1I). These results indicated that obesity exacerbated the inflammatory injuries of SAP, with ATMs potentially mediating this process.

### 2.2. The Autophagic Flux Was Impaired in ATMs After the Induction of Obesity-Related SAP

**Figure 2:**
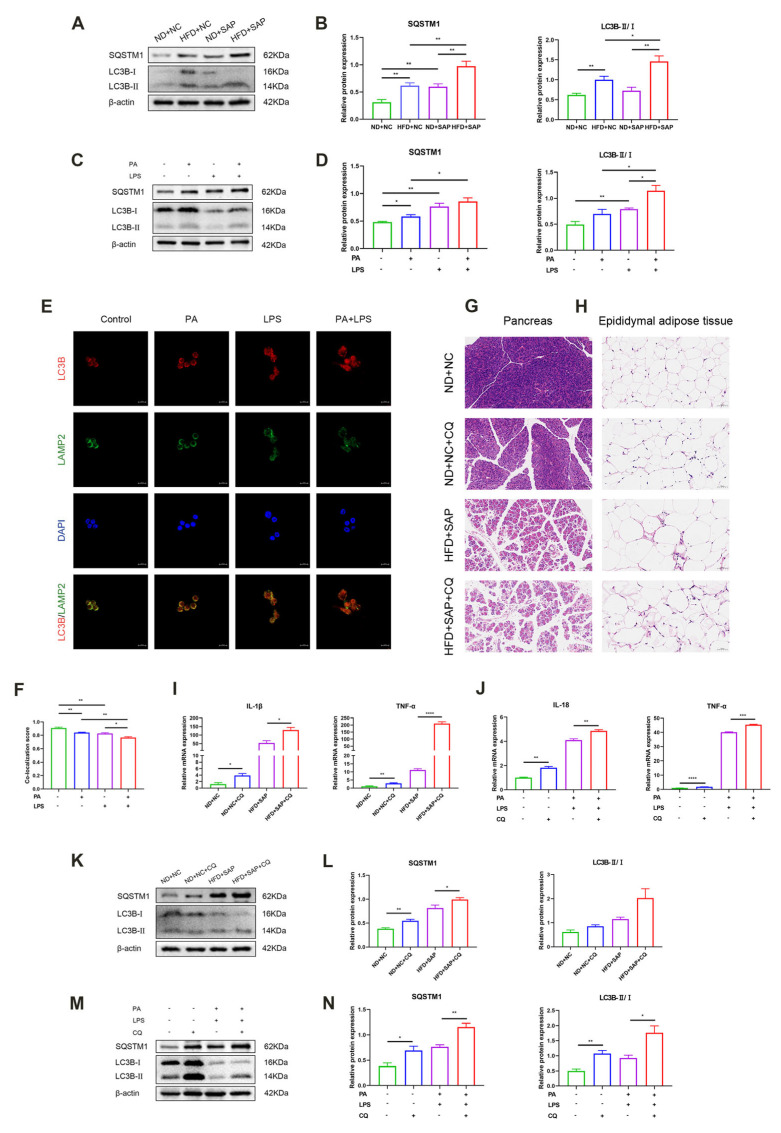
The autophagic flux was impaired in ATMs after the induction of obesity-related SAP. Western blot for the expression of SQSTM1/p62 and LC3B in adipose tissue (**A**) and relative protein expressions (**B**). Western blot for the expression of SQSTM1/p62 and LC3B in macrophages (**C**) and relative protein expressions (**D**). (**E**) Immunofluorescence images for LC3B and LAMP2 in macrophages (scale 10 μm). (**F**) Co-localization score of LC3B and LAMP2. HE staining of the pancreatic tissue (**G**); (scale 100 μm) and epididymal adipose tissue (**H**); (scale 50 μm) after CQ intervention. (**I**) The mRNA levels of IL-1β and TNF-α in adipose tissue were tested via qPCR. (**J**) The mRNA levels of IL-18 and TNF-α in macrophages were examined via qPCR. Western blot for the expression of SQSTM1/p62 and LC3B in adipose tissue (**K**) and relative protein expressions (**L**). Western blot for the expression of SQSTM1/p62 and LC3B in macrophages (**M**) and relative protein expressions (**N**). * *p* < 0.05, ** *p* < 0.01, *** *p* < 0.001, and **** *p* < 0.0001.

The change in autophagic flux in adipose tissue was examined by Western blot first (Figure 2A,B). Sequestosome-1 (SQSTM1)/p62 and microtubule associated protein 1 light chain 3 beta (LC3B) are the markers of autophagic cargo and autophagosome, respectively. The results showed the significant accumulation of SQSTM1/p62 and LC3B after the induction of obesity-related SAP, suggesting impaired autophagic flux. Experiments in vitro showed that the stimulation of PA and LPS reduced the autophagic flux in macrophages (Figure 2C,D). Immunofluorescence showed decreased co-expression of LC3B and lysosomal associated membrane protein 2 (LAMP2) in macrophages after the stimulation of PA and LPS (Figure 2E,F). This indicated a decline in autophagosome–lysosome fusion, which could block the autophagic flux. To establish the pathological relevance of autophagic flux impairment, chloroquine (CQ), an autophagy inhibitor [20], was applied.

CQ administration exacerbated inflammatory injuries of pancreas and adipose tissue (Figure 2G,H). CQ treatment elevated inflammatory cytokine levels in adipose tissue (Figure 2I). Experiments in vitro confirmed the effect of CQ to promote inflammatory macrophages (Figure 2J). Supplementary Western blot demonstrated CQ treatment induced the accumulation of SQSTM1/p62 and LC3B (Figure 2K,L), which was similar to the impairment pattern observed during SAP induction. The experiments in vitro further confirmed this (Figure 2M,N).

### 2.3. Expression of Autophagosome-Localized SNARE Proteins Decreased in ATMs of Obesity-Related SAP

**Figure 3:**
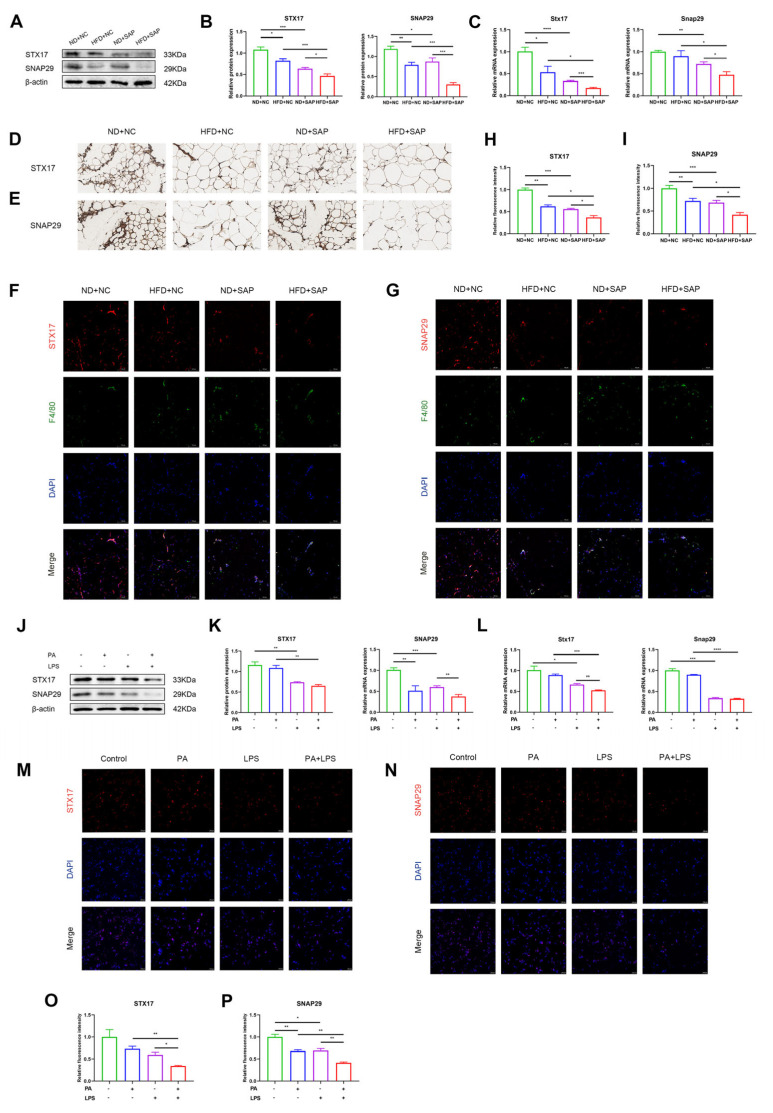
Expression of autophagosome-localized SNARE proteins decreased in ATMs of obesity-related SAP. Western blot for the expression of STX17 and SNAP29 in adipose tissue (**A**) and relative protein expressions (**B**). (**C**) The mRNA levels of Stx17 and Snap29 in adipose tissue were examined via qPCR. Immunohistochemistry for STX17 (**D**) and SNAP29 (**E**) in adipose tissue (scale: 50 μm). Immunofluorescence images for F4/80 and STX17 (**F**) or SNAP29 (**G**) in adipose tissue (scale: 100 μm). Relative average fluorescence intensity of STX17 (**H**) and SNAP29 (**I**) in F4/80^+^ cells. Western blot for the expression of STX17 and SNAP29 in macrophages (**J**) and relative protein expressions (**K**). (**L**) The mRNA levels of Stx17 and Snap29 in macrophages were tested via qPCR. Immunofluorescence images for STX17 (**M**) and SNAP29 (**N**) in macrophages (scale: 100 μm). Relative average fluorescence intensity of STX17 (**O**) and SNAP29 (**P**) in macrophages. * *p* < 0.05, ** *p* < 0.01, *** *p* < 0.001, and **** *p* < 0.0001.

The SNARE proteins connect autophagosome and lysosome to mediate the fusion. Among the SNAREs, autophagosome-localized Q-SNAREs, marked as syntaxin 17 (STX17) and synaptosomal associated protein 29 (SNAP29), are involved in the process of fusion [19]. Western blot and qPCR analyses demonstrated that both the induction of obesity and SAP reduced the protein or mRNA levels of STX17 and SNAP29 in adipose tissue (Figure 3A–C). The immunohistochemistry of adipose tissue showed decreased STX17 and SNAP29 puncta in obesity-related SAP mice (Figure 3D,E). Immunofluorescence for STX17 or SNAP29 co-staining with F4/80 indicated a lower average expression of STX17 or SNAP29 in ATMs of the obesity-related SAP group (Figure 3F–I). A subsequent analysis of Q-SNARE proteins in pretreated macrophages was performed. The results of Western blot and qPCR showed that the stimulation of PA and LPS could reduce the levels of STX17 and SNAP29 (Figure 3J–L). The immunofluorescence of macrophages also indicated decreased expression of Q-SNARE proteins (Figure 3M–P).

### 2.4. Expression and Transcriptional Activity of FoxO1 Decreased in Obesity-Related SAP

**Figure 4:**
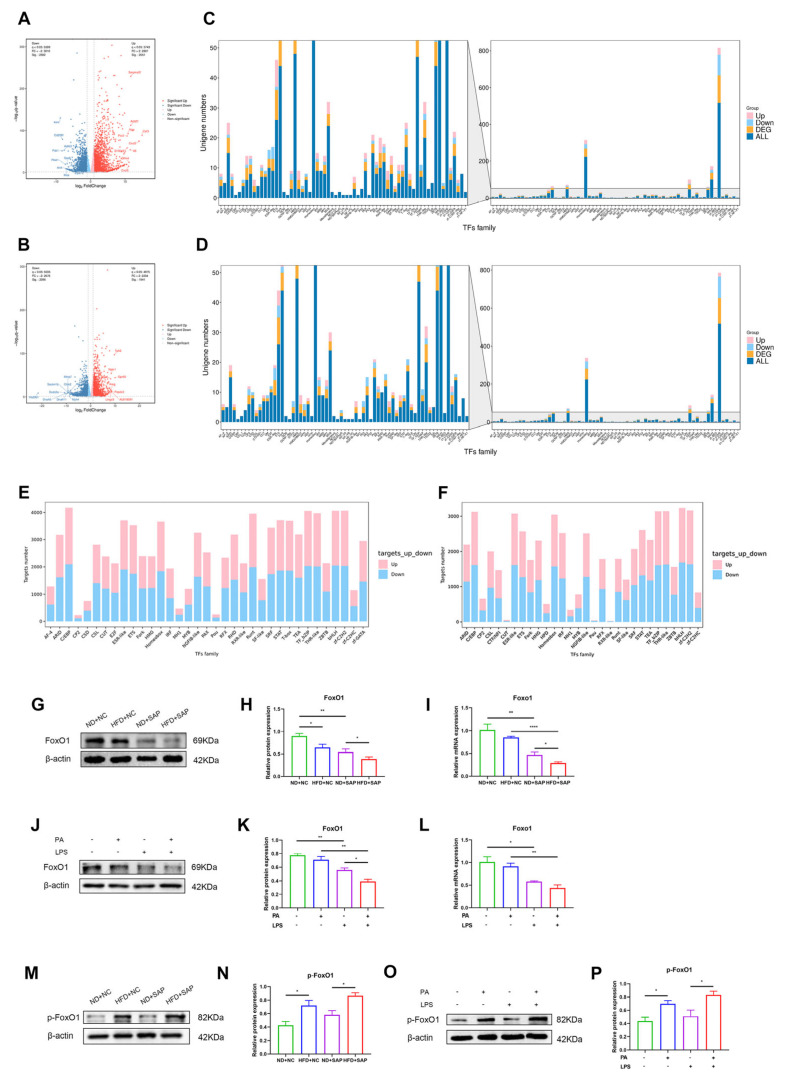
Expression and transcriptional activity of FoxO1 decreased in obesity-related SAP. Volcano maps of DEGs of the HFD+SAP vs. HFD+NC groups (**A**) and of the HFD+SAP vs. ND+SAP groups (**B**). Differentially expressed transcription factors of the HFD+SAP vs. HFD+NC groups (**C**) and of the HFD+SAP vs. ND+SAP groups (**D**). Differentially expressed targeted genes of the HFD+SAP vs. HFD+NC groups (**E**) and of the HFD+SAP vs. ND+SAP groups (**F**). Western blot for the expression of FoxO1 in adipose tissue (**G**) and relative protein expressions (**H**). (**I**) The mRNA levels of Foxo1 in adipose tissue were examined via qPCR. Western blot for the expression of FoxO1 in macrophages (**J**) and relative protein expressions (**K**). (**L**) The mRNA levels of Foxo1 in macrophages were examined via qPCR. Western blot for the expression of p-FoxO1 in adipose tissue (**M**) and relative protein expressions (**N**). Western blot for the expression of p-FoxO1 in macrophages (**O**) and relative protein expressions (**P**). * *p* < 0.05, ** *p* < 0.01, and **** *p* < 0.0001.

The potential regulatory factor of Q-SNARE proteins in obesity-related SAP was further studied through RNA-sequencing (RNA-seq). The volcano plots showed differentially expressed genes (DEGs) in the HFD+SAP group compared with the HFD+NC group and the ND+SAP group (Figure 4A,B). The DEGs were subsequently annotated to transcription factor (TF) families and were reviewed (Figure 4C,D). By comprehensively comparing the HFD+SAP group with the HFD+NC group and the ND+SAP group, Forkhead box (Fork) TFs were recognized among the TF families containing the most DEGs, as Fork TFs were highly associated with autophagy [21]. A further analysis also indicated alterations in targeted genes of Fork TFs in obesity-related SAP, suggesting that the transcriptional activity changes (Figure 4E,F). The expression of Forkhead box O1 (FoxO1), the most representative transcription factor of Fork TFs, was further examined. Western blot (Figure 4G,H) and qPCR (Figure 4I) showed that the induction of obesity-related SAP significantly decreased the expression of FoxO1 in adipose tissue. Experiments in vitro also showed that PA and LPS stimulation inhibited the expression of FoxO1 in macrophages (Figure 4J–L). Furthermore, Phospho-FoxO1 (p-FoxO1) increased in vivo and in vitro as the expression of FoxO1 decreased (Figure 4M–P). Given that phosphorylation promotes FoxO1 nuclear exclusion [22], this finding suggested the possible decreased transcriptional activity of FoxO1.

### 2.5. FoxO1 Regulated the Expression of Autophagosome-Localized SNARE Proteins in ATMs

**Figure 5:**
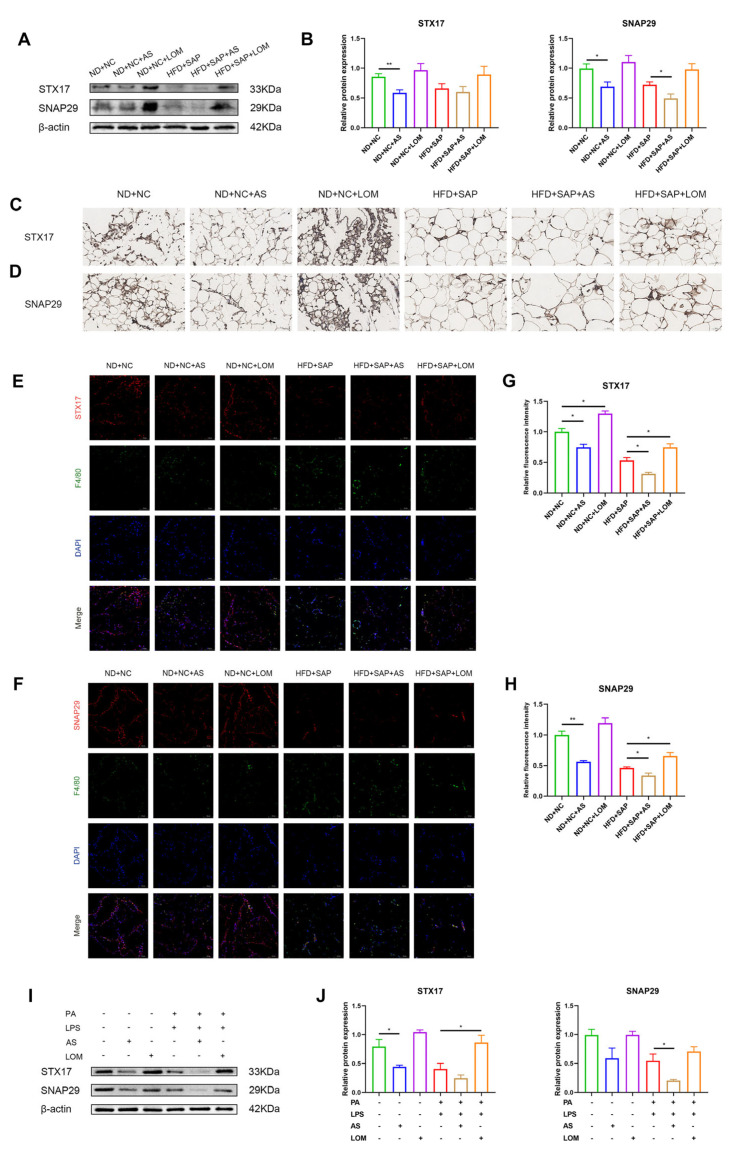
FoxO1 regulated the expression of autophagosome-localized SNARE proteins in ATMs. Western blot for the expression of STX17 and SNAP29 in adipose tissue (**A**) and relative protein expressions (**B**) after the intervention of AS and LOM. Immunohistochemistry for STX17 (**C**) and SNAP29 (**D**) in adipose tissue (scale: 50 μm). Immunofluorescence images for F4/80 and STX17 (**E**) or SNAP29 (**F**) in adipose tissue (scale: 100 μm). Relative average fluorescence intensity of STX17 (**G**) and SNAP29 (**H**) in F4/80^+^ cells. Western blot for the expression of STX17 and SNAP29 in macrophages (**I**) and relative protein expressions (**J**). * *p* < 0.05, ** *p* < 0.01.

To test the regulatory effect of FoxO1 on Q-SNARE proteins, AS1842856 (AS), a specific FoxO1 inhibitor, and LOM612 (LOM), a specific FoxO1 activator, were applied. AS mainly affects the transcriptional activity of FoxO1 [23], while LOM acts on the nuclear translocation [24]. Western blot showed that AS treatment could reduce the expression of STX17 and SNAP29. Conversely, the expression of STX17 and SNAP29 increased after LOM administration (Figure 5A,B). Immunohistochemistry confirmed these trends of STX17 and SNAP29 (Figure 5C,D). The immunofluorescence of adipose tissue for STX17 or SNAP29 co-staining with F4/80 demonstrated the altered expressions in ATMs (Figure 5E–H). These results indicated that FoxO1 could regulate the expression of STX17 and SNAP29. Western blot and qPCR in vitro showed AS stimulation could inhibit the expression of STX17 and SNAP29 in pretreated macrophages, while LOM stimulation could promote the expressions (Figure 5I,J).

### 2.6. FoxO1 Regulated the Inflammation and Autophagic Flux in ATMs

**Figure 6:**
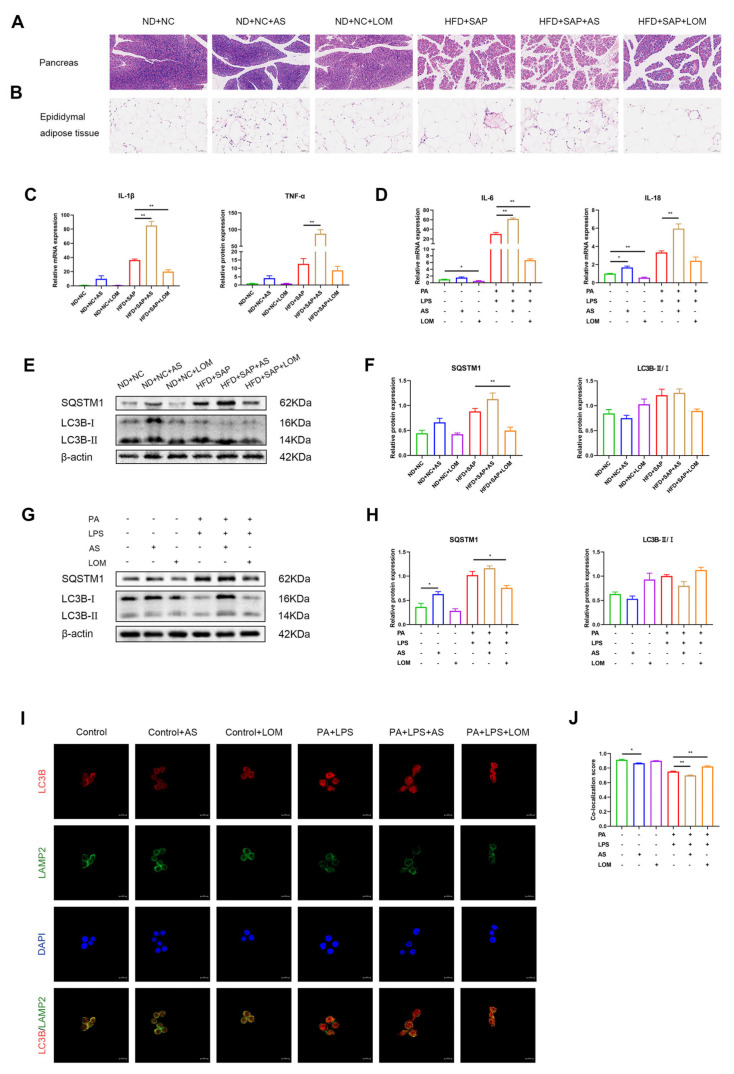
FoxO1 regulated the inflammation and the autophagic flux in ATMs. HE staining of the pancreatic tissue (**A**) (scale: 100 μm) and epididymal adipose tissue (**B**) (scale: 50 μm) after the intervention of AS and LOM. (**C**) The mRNA levels of IL-1β and TNF-α in adipose tissue were examined via qPCR. (**D**) The mRNA levels of IL-6 and IL-18 in macrophages were examined via qPCR. Western blot for the expression of SQSTM1/p62 and LC3B in adipose tissue (**E**) and relative protein expressions (**F**). Western blot for the expression of SQSTM1/p62 and LC3B in macrophages (**G**) and relative protein expressions (**H**). (**I**) Immunofluorescence images for LC3B and LAMP2 in macrophages (scale: 10 μm). (**J**) Co-localization score of LC3B and LAMP2. * *p* < 0.05, ** *p* < 0.01.

The inflammatory injuries of the pancreas and adipose tissue after the administration of AS and LOM were evaluated. Compared with the obesity-related SAP group, AS-treated mice exhibited exacerbated inflammatory injury, while LOM administration attenuated inflammation severity (Figure 6A,B). A qPCR analysis of adipose tissue showed upregulated inflammatory cytokine levels in AS-treated mice and downregulated levels with LOM exposure (Figure 6C). Experiments in vitro further confirmed the trends in macrophages (Figure 6D). Autophagic flux in adipose tissue was further examined through Western blot (Figure 6E,F). AS administration increased the accumulation of SQSTM1/p62 in obesity-related SAP mice, which was reduced after LOM administration. Consistent with in vivo observations, the Western blot of macrophages confirmed AS-induced SQSTM1/p62 accumulation and its downregulation via LOM (Figure 6G,H). Immunofluorescence further showed decreased co-expression of LC3B and LAMP2 in the AS group, while LOM enhanced the co-expression (Figure 6I,J). These results suggested the FoxO1-mediated regulation of SAP inflammation through the modulation of autophagic flux.

## 3. Discussion

Obesity, a significant and growing health concern, has been identified as a primary risk factor for adverse outcomes in SAP. Notably, obesity-related SAP exhibits a distinct pathological feature characterized by the excessive infiltration of ATMs, which account for 40–50% of adipose cellular components in advanced obesity [25]. Our previous studies indicated that ATMs polarized to M1 phenotype and secreted inflammatory cytokines in obesity-related SAP [11,26]. The current study confirmed the aggravated inflammation of obesity-related SAP and demonstrated that macrophages within lipid-enriched inflammatory microenvironments could exacerbate inflammatory damage of pancreatic acinar cells.

Although the pathogenesis of AP remains incompletely understood, autophagy, a lysosomal-dependent degradation pathway, has been demonstrated to be involved in the development of AP. Lysosomes execute the terminal phase of autophagy by fusing with autophagosomes and degrading the autophagic cargo. Previous studies have observed an insufficient autophagic flux, due to impaired lysosome–autophagosome fusion, in the pancreas during AP [27,28]. Intriguingly, similar autophagic impairment has been documented in high-fat-diet-fed mouse models [29]. In this study, we investigated the autophagic flux within adipose tissue. The results showed that SQSTM1/p62 and LC3B, the markers of autophagic cargo and autophagosomes, accumulated in obesity-related SAP. Experiments in vitro using macrophages exposed to lipid overload and inflammatory stimulation showed the same accumulation accompanied by a decreased co-expression of LAMP2 and LC3B, suggesting impairments of autophagic flux and lysosome–autophagosome fusion in ATMs. The pharmacological inhibition of autophagy with CQ that prevents autophagosome–lysosome fusion exacerbated inflammatory responses in vitro and in vivo. We proposed that ATMs with impaired autophagic flux likely contribute to the aggravated inflammatory injuries of obesity-related SAP.

Maintaining the quantity and quality of lysosomes, as well as efficient lysosome–autophagosome fusion, is critical for sufficient autophagic flux. While dysfunctional and decreased lysosomes have been reported in pancreatitis models [30,31], the other regulatory mechanisms of lysosome–autophagosome fusion in AP remain poorly understood. The role of SNARE proteins as mediators of autophagosome–lysosome fusion is well established. Autophagosome-localized STX17 or SNAP29 and lysosome-localized vesicle-associated membrane protein 8 (VAMP8) or vesicle-associated membrane protein 7 (VAMP7) are believed to be the key factors involved in this process [19,32]. Due to the limited relevant research, this study mainly focused on the alterations of autophagosome-localized SNARE proteins in obesity-related SAP. The results showed that the expression of STX17 and SNAP29 in adipose tissue significantly decreased after the induction of obesity and SAP. Further validation in vitro showed reduced Q-SNARE proteins in macrophages under the stimulation of PA and LPS. These findings indicated the dysfunction of autophagosome-localized SNARE proteins in ATMs during obesity-related SAP, which could lead to the impaired autophagic flux.

In the current study, we performed RNA-seq to identify the potential regulatory pathway underlying the impaired autophagic flux and SNARE proteins. By comparing the obesity-related SAP group with the normal-diet SAP group or the obesity-control group, DEGs were observed in Forkhead transcription factors, as well as the differentially expressed targeted genes. FoxO belongs to the family of Forkhead transcription factors. Notably, FoxO transcription factors have been shown to regulate metabolic homeostasis and autophagy [21,33,34]. Previous study showed deletion of FoxO1 dysregulated lipid metabolism and downregulated autophagy [35]. Some evidence suggested that FoxO1 could also mediate autophagy by regulating other factors such as autophagy-related gene 7 (ATG7) and RAB7 [36,37]. Obesity and acute pancreatitis are both pathological processes related to lipid metabolism [38,39,40]. Considering that it is associated with both lipid metabolism and autophagy, FoxO1 was recognized as the possible regulator. A further examination in the current study demonstrated a decreased expression of FoxO1 in obesity-related SAP. We also found that phosphorylated FoxO1 increased after the induction of obesity-related SAP. Since FoxO1 phosphorylation promotes its nuclear exclusion, this finding indicated that the transcription activity of FoxO1 also decreased. The effects of FoxO1 on SNARE proteins and autophagy were subsequently examined. The results showed that the inhibition of FoxO1 reduced the expression of STX17 and SNAP29, while the activation of FoxO1 showed the opposite effect. Furthermore, the inhibition of FoxO1 aggravated the impaired autophagic flux in ATMs during obesity-related SAP and contributed to more severe inflammatory injuries.

There were several limitations to this study. First, this study was mainly based on mouse models. The results require further validation from clinical specimens. Second, previous studies indicated that autophagy could regulate apoptosis or pyroptosis [41,42]. The intricate interplay between autophagy and apoptosis or pyroptosis in obesity-related SAP will be further studied. Third, the possible regulatory effect of FoxO1 on lysosome-localized SNARE proteins and other autophagic factors in obesity-related SAP needs further investigation.

In summary, the impaired autophagic flux in ATMs contributed to exacerbated inflammatory injuries during obesity-related SAP. FoxO1 mediated the impaired autophagic flux by regulating the expression of autophagosome-localized SNARE proteins. This FoxO1-SNARE proteins axis represented a possible link between metabolic dysregulation and inflammatory amplification in obesity-related SAP.

## 4. Materials and Methods

### 4.1. Animal Grouping and Processing

Four-week-old male C57BL/6 mice were obtained from Zhuhai Bestest Biotechnology (Zhuhai, Guangdong, China). The mice were randomly assigned to four groups: a normal-diet control group (ND+NC), a normal-diet SAP group (ND+SAP), a high-fat-diet control group (HFD+NC), and a high-fat-diet SAP group (HFD+AP). Mice in the normal-diet groups were fed standard diets (fat kcal%: 12.11%; Guangdong Medical Laboratory Animal Center, Guangzhou, Guangdong, China). Mice in the high-fat-diet groups were fed high-fat diets (fat kcal%: 60%; Ready Dietech, Guangzhou, Guangdong, China) to induce obesity. All animals were maintained on their respective diets for 16 weeks. SAP was induced via an intraperitoneal injection of caerulein (50 µg/kg, seven times, one-hour interval; MCE, NJ, USA), followed by LPS (10 mg/kg; MCE). The control groups received normal saline. Subsets of mice in the ND+NC and HFD+SAP groups received an intraperitoneal injection of chloroquine (60 mg/kg; MCE) before caerulein injection. The other two subsets of mice in the ND+NC and HFD+SAP groups received an intraperitoneal injection of AS1842856 (10 mg/kg; MCE) or LOM612 (10 mg/kg; MCE) before a caerulein injection. Twenty-four h after LPS injection, the mice were euthanized. Serum samples, the pancreas, and epididymal adipose tissue were collected for further analysis. The animal experiments were approved by the Institutional Animal Care and Use Committee of Sun Yat-Sen University (SYSU-IACUC-2024-002158).

### 4.2. Histopathologic Assessment

The collected tissues were fixed in paraformaldehyde and subsequently embedded in paraffin. The paraffin-embedded tissue sections were stained with hematoxylin/eosin (HE) and examined using an optical microscope. The histopathological scoring of pancreatic tissue was based on the presence of edema, acinar necrosis, inflammatory infiltration, hemorrhage, and fat necrosis [43].

### 4.3. Biochemical Assays

Serum samples were collected for biochemical assays. The levels of serum lipase and amylase were measured using assay kits according to the instructions of the manufacturer (Nanjing Jiancheng Bioengineering Institute, Nanjing, Jiangsu, China).

### 4.4. Cell Culture of RAW264.7 Macrophages and 266-6 Pancreatic Acinar Cells

Murine-derived Raw264.7 macrophages (Pricella, Wuhan, Hubei, China) and 266-6 pancreatic acinar cells (Bohui Biotechnology, Guangzhou, Guangdong, China) were cultured in Dulbecco’s modified eagle medium (DMEM, Gibco, CA, USA) supplemented with 10% bovine calf serum at 37 °C and 5% CO_2_. To simulate the lipid condition of adipose tissue macrophages, PA (100 μM; MCE), an effector of lipid injury [44], was used to stimulate Raw264.7 cells. The control groups were treated with acid-free bull serum albumin. LPS (0.1 μg/mL; MCE) was used to simulate the acute inflammatory condition. Macrophages were cultured for 12 h before further analysis. In addition, macrophages were added onto a porous 0.4-uM polyester membrane transwell insert with the treatment of PA and LPS for 12 h. Then pancreatic acinar cells were co-cultured with macrophages through the transwell system for another 12 h and collected for analysis. Chloroquine (50 μM, MCE), AS1842856 (1 μM, MCE), or LOM612 (1 μM, MCE) was used to intervene macrophages prior to treatment of PA and LPS in specific experiments.

### 4.5. Cell Viability Assay with Cell Counting Kit-8

The transwell system was removed after the co-culture of macrophages and pancreatic acinar cells. The cell viability of pancreatic acinar cells was examined using Cell Counting Kit-8 (CCK-8) kits according to the instructions of the manufacturer (NCM Biotech, Suzhou, Jiangsu, China).

### 4.6. RNA Analysis

Total RNA was extracted from adipose tissue samples and RAW264.7 cells using RNAiso Plus (Takara, Kyoto, Japan). cDNA synthesis was performed using the cDNA synthesis kit (AG, Changsha, Hunan, China). qPCR was conducted using SYBR Green Premix kit (AG) on a Roche LightCycler^®^ 480 II PCR system.

### 4.7. Western Blot

Proteins were extracted from adipose tissue samples and RAW264.7 cells using a lysis buffer (Beyotime, Shanghai, China) containing the protease inhibitor (Beyotime). A Western blot analysis was performed using the protein lysates. Membranes were incubated with primary antibodies overnight, followed by incubation with secondary antibodies. The immunoblots were visualized using an enhanced chemiluminescence system. The following antibodies were used: FoxO1 antibody (1:1000 dilution; Abcam, Cambridge, UK), Phospho-FoxO1 (Ser256) antibody (1:1000 dilution; CST, Boston, MA, USA), STX17 antibody (1:1000 dilution; Abcam), SNAP29 antibody (1:1000 dilution; Proteintech, Wuhan, Hubei, China), SQSTM1/p62 antibody (1:1000 dilution; Abcam), LC3B antibody (1:1000 dilution; Proteintech), Beta-Actin antibody (1:4000 dilution; Proteintech), and Goat Anti-Rabbit IgG (1:2000 dilution; Proteintech).

### 4.8. Immunofluorescence

Adipose tissue sections were incubated with an F4/80 antibody (1:200 dilution; MCE) and an STX17 antibody (1:200 dilution; Abcam) or a SNAP29 antibody (1:200 dilution; Proteintech). The sections were then incubated with fluorescence-labeled secondary antibodies and counterstained with DAPI (Servicebio, Beijing, China). The pretreated macrophages were also incubated with the STX17 antibody (1:100 dilution), the SNAP29 antibody (1:100 dilution), or the LC3B antibody (1:100 dilution; Proteintech) and the LAMP2 antibody (1:100 dilution; Proteintech), followed by incubation with secondary antibodies and DAPI. The samples were visualized and photographed using a fluorescence microscope (Leica, Wetzlar, German; ZEISS, Oberkochen, German).

### 4.9. Immunohistochemistry

Adipose tissue sections were deparaffinized and rehydrated. Sections were then incubated with the STX17 antibody (1:200 dilution; Abcam) or the SNAP29 antibody (1:200 dilution; Proteintech) overnight. Subsequently, sections were incubated with the HRP-labeled Goat Anti-Rabbit secondary antibody and diaminobenzidine for color development. Images were acquired using an optical microscope.

### 4.10. RNA-Sequencing

Total RNA was extracted using the mirVana RNA Isolation Kit (Ambion, Austin, TX, USA) following the manufacturer’s protocol. RNA integrity was evaluated using the Agilent 2100 Bioanalyzer (Agilent Technologies, Santa Clara, CA, USA). The libraries were constructed using TruSeq Stranded Total RNA with Ribo-Zero Gold (Illumina, San Diego, CA, USA), according to the manufacturer’s instructions. Then, these libraries were sequenced on the Illumina sequencing platform (HiSeqTM 2500, Illumina). DEGs were screened by criteria of fold-change > 2 or fold-change < 0.5 and *p*-value < 0.05.

### 4.11. Graphical Depiction and Statistics

The data were presented as means ± SEMs. Differences between groups were evaluated using a *t*-test or an ANOVA. A *p*-value < 0.05 was considered statistically significant. The statistical analysis and graphical presentations were performed using the GraphPad Prism software (version 8.0).

## Data Availability

The original contributions presented in this study are included in the article. Further inquiries can be directed to the corresponding authors.
